# X-Ray Fluorescence Ionomics of Herbarium Collections

**DOI:** 10.1038/s41598-019-40050-6

**Published:** 2019-03-18

**Authors:** Antony van der Ent, Guillaume Echevarria, A. Joseph Pollard, Peter D. Erskine

**Affiliations:** 1 Centre for Mined Land Rehabilitation, Sustainable Minerals Institute, The University of Queensland, Queensland, Australia; 2 Laboratoire Sols et Environnement, Université de Lorraine, Nancy, France; 3 Department of Biology, Furman University, Greenville, South Carolina USA

## Abstract

Global herbaria are the greatest repositories of information on the plant kingdom. Discoveries of trace element hyperaccumulator plants have historically required time-consuming destructive chemical analysis of fragments from herbarium specimens, which severely constrains the collection of large datasets. Recent advances in handheld X-Ray Fluorescence spectroscopy (XRF) systems have enabled non-destructive analysis of plant samples and here we propose a new method, which we term “Herbarium XRF Ionomics”, to extract elemental data from herbarium specimens. We present two case studies from major tropical herbaria where Herbarium XRF Ionomics has led to the discovery of new hyperaccumulator plants and provided valuable insights into phylogenetic patterns of trace element hyperaccumulation. Herbarium XRF Ionomics is a new value proposition for continued funding and retention of herbarium specimens globally.

## Introduction

Plants take up macronutrients (Ca, K, Mg, P, S), micronutrients (Fe, B, Cl, Cu, Mn, Mo, Ni, Zn), elements that are beneficial to some plants (Al, Na, Co, Se, Si), and also non-essential trace elements (As, Cd, Hg, Pb, Se, Tl)^1^. The supply of essential elements to a plant ranges from deficiency to optimum and eventual toxicity, and differs greatly between elements^2^. The elemental composition of plant tissues is controlled by genetically predisposed ecophysiological behaviour, and the availability of specific elements in the soil, as well as other environmental factors (climate, water supply, altitude, etc.). Taken together the concentrations of elements in plant tissue make up the “ionome^3,4^”. Ionomics has the ability to capture information about the physiology of a plant under different conditions, driven by genetic differences and environmental filters^4,5,6^. The ionome (or foliar elemental profile) can provide a wide range of information on: (i) phylogenetic patterns of trace elemental accumulation and nutrient acquisition; (ii) geographic and geological occurrence of minerals, including those of commercial value; and (iii) elemental homeostasis, including extremes of uptake, co-accumulation or exclusion of elements, in response to soil and habitat variation.

Plant species that accumulate trace elements to extreme concentrations are known as hyperaccumulators^7–9^. The phenomenon of hyperaccumulation is interesting from an evolutionary and physiological viewpoint, and also finds practical application in phytomining and related novel technologies^10,11^. Globally the discovery of hyperaccumulator plants has been hindered by the lack of systematic screening of plant species from different phylogenetic lineages, and is highly biased towards specific regions around the world, *e.g*. ultramafic regions^8,9^. Furthermore, most effort and focus has been on Ni hyperaccumulator plants, due in part to the existence of a simple reagent paper test (based on dimethylglyoxime), such that Ni accounts for over 500 of the approximately 700 known hyperaccumulator species^12^. However, this does not necessary mean that hyperaccumulators of other elements, such as Mn or Zn, are rare. It may merely mean that they have not yet been discovered.

The value of herbaria as references for taxonomic, genetic and biogeographic information is widely acknowledged. However, herbaria may also render other sources of information. Here we propose the “Herbarium XRF Ionomics” approach to extract new elemental data from herbarium specimens using X-ray fluorescence spectroscopy (XRF). Herbarium XRF Ionomics can provide insights into phylogenetic and biogeographic patterns of trace element accumulation by plants for a wide range of different chemical elements. New technology allows the process to be conducted rapidly, with no damage to herbarium specimens, and therefore gains access to this hitherto untapped resource of information.

**Figure 1:**
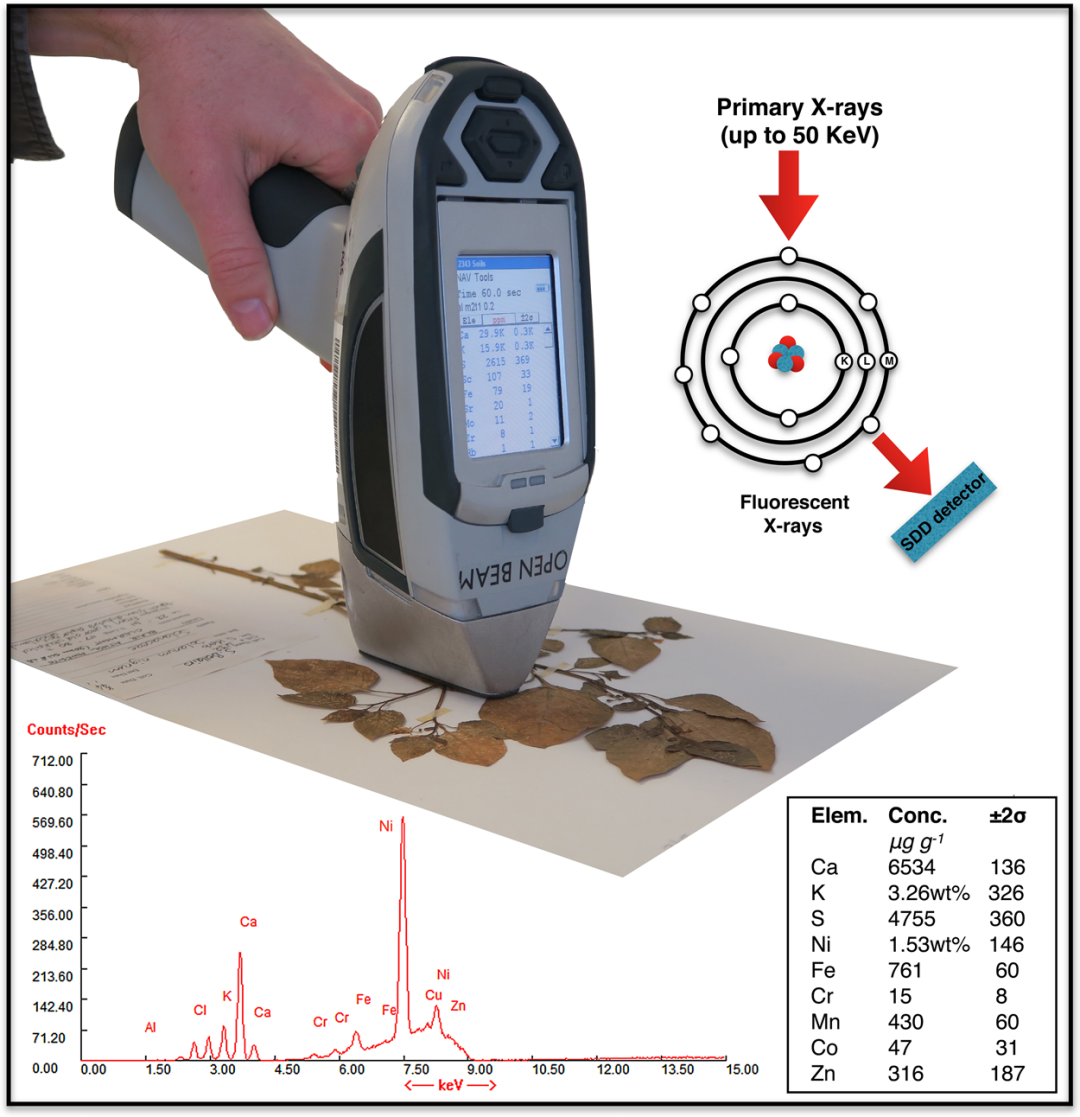
Measurement of herbarium specimen using handheld X-Ray Fluorescence analyser (ThermoFisher Scientific Niton XL3t 950). Manufacturer branding and logo has been digitally removed. For details, see Methods.

In order to understand how foliar elements are regulated and how this relates to the ecophysiology of a plant species, it is necessary to measure as many elements as possible on a large number of specimens^13^. Therefore, ionomics requires high-throughput elemental analysis technologies and their integration with bioinformatics^4^. Recently, handheld XRF systems have been validated for measuring plant nutrients in agronomic samples, although this initially involved powdering and pelletisation of the plant material for analysis^14–16^. The advent of portable XRF instruments utilizing the latest type of fast detectors now enables non-destructive analysis of plant material and permits mass measurements of tens of thousands of samples in a relatively short time span at low cost. The latest generation of XRF instruments (equipped with Ag or Rh anode operating at 50 kV, 200 µA) can measure the concentrations of a wide range of different elements in a spot of ~6 mm in under two minutes with a detection limit of ~100 μg g^−1^ for most of the transition elements (such as Ni, Co, Zn). As such, a single operator can measure herbarium specimens at a rate of >300 specimens per day. For the first time, it is now feasible to determine elemental concentrations across entire phylogenetic lineages, including many replicate specimens of the same species orginating from different collection localities. Additionally, XRF screening (Fig. 1) may be combined with the (photographic) digitisation process of herbarium specimens, an effort already underway in many herbaria worldwide.

Given that XRF devices are designed for measuring elemental concentrations in rock and soil matrices, validation for dried plant leaves (essentially a 200–500 micron thick cellulose low *Z* element matrix), is necessary. As detailed in the Methods section, Compton Normalisation in combination with element-specific emperical correction factors can be used for calibration of the X-ray fluorescence data. Correction factors must be determined empirically by measuring a selection of non-herbarium samples using both XRF and more traditional, destructive analytical methods such as inductively coupled plasma optical emission spectroscopy (ICP-OES) on acid-digested plant samples. Soil dust contamination adhering to leaves can confound measurements, but may be assessed from unusually high concomitant Cr, Fe, and Ti concentrations^17^. Another issue is the practise of some tropical collectors to use methylated spirit for temporarily preserving sample collections to prevent decomposition of the specimens during transport, which can potentially leach soluble elements from the plant material or contaminate other specimens; thus, such specimens should be avoided when possible. Finally, some (old) herbarium specimens may be treated with HgCl_2_ for long-term insect protection and therefore readings for Hg can be extremely high for such samples (*i.e*. > 500 μg g^−1^).

## Results and Discussion

Brief summaries of two case studies of the application of Herbarium XRF Ionomics are summarized here, to provide an indication of the potential of this approach.

The Malaysian state of Sabah on the island of Borneo has high levels of plant diversity (>8000 species), occurring on a wide range of soil types^18^. Sabah has over 3500 km^2^ of ultramafic surface geology (~4.6% of the total landmass of the state), forming soils with high concentrations of Mg, Ni, Co, Cr, Cu, and Mn. More than 4250 plant species naturally occur on this substrate, including many known hyperaccumulators^18,19^. This makes it one of the most species-rich floras occupying ultramafic outcrops globally^20^. Using XRF scanning, ~7300  herbarium specimens were analysed at the Forest Research Centre (FRC) in Sabah, Malaysia. The measurements recorded 12 Co hyperaccumulator species (in 3 families, 7 genera), 51 Mn hyperaccumulator species (in 12 families, 24 genera) and 28 Ni hyperaccumulator species (in 10 families, 17 genera). Prior to the XRF scanning 3 Co hyperaccumulator species, 7 Mn hyperaccumulator species, 24 Ni hyperaccumulator species, and 2 Zn hyperaccumulator species,   were known from Sabah^18,21,22^. The discovery of Zn hyperaccumulators here was unexpected, as previously-known Zn hyperaccumulators are from temperate climate regions and herbaceous species^9^. Our new approach also helps to explain the scientific oddity *Dichapetalum gelanioides*, recorded twenty-five years ago^18^ with a sub-species (subsp. *tuberculatum*) that hyperaccumulates Ni on ultramafic soils and other sub-species (subsp. *pilosum* and subsp. *sumatranum*) hyperaccumulating Zn on ‘normal’ non-Zn-rich soils. Extraordinarily this species appears to hyperaccumulate Zn from non-mineralised soils with only background concentrations of Zn^23^. This raises important questions about the evolution of both Ni and Zn hyperaccumulation: did Ni-hyperaccumulation evolve when species that were already Zn-hyperaccumulators on non-ultramafic soils colonised ultramafic soils? Targeted herbarium XRF screening has already yielded other fascinating discoveries, including Co hyperaccumulation in a tree from Borneo^24^, and Ni hyperaccumulation in a critically endangered species from a genus (*Antidesma*; Phyllanthaceae) previously unknown to contain hyperaccumulator plants^25^.

New Caledonia, in the southwest Pacific Ocean, is an island biodiversity hotspot harbouring over 3371 vascular plant species, of which nearly 75% are endemic^26^. The New Caledonian vegetation over ultramafic rocks contains 2145 species, including 1747 (80%) endemics^27,28^. The ultramafic flora and hyperaccumulators from New Caledonia are probably the best studied in the world^27^. New Caledonia is a global hotspot for hyperaccumulator plants, with 65 nickel and 11 manganese hyperaccumulator species recorded to date^27,29^. XRF analysis at the Institute for Research and Development (IRD) Herbarium in Nouméa, New Caledonia examined ~11 200  herbarium specimens (in 35 orders, 96 families, 281 genera, 1620 taxa). The sampling effert covered plant families known to contain numerous hyperaccumulators (*e.g*. Cunoniaceae, Phyllanthaceae, Salicaceae, Sapotaceae and Violaceae), as well as a systematic screening of 1–4 specimens (depending on availability in the herbarium) of all  species known to occur on ultramafic soils in New Caledonia. The latter group of specimens (1372 species) represented 88.5% of the known taxa of vascular plants occurring on ultramafic outcrops in New Caledonia. Numerous marginal hyperaccumulator plants for Ni, Mn, Co, and Zn were noted; however, the results summarised here are restricted to exceptionally high records (*i.e*. Ni > 5000 µg g^−1^, Mn > 20 000 µg g^−1^, Co > 1000 µg g^−1^, and Zn > 10 000 µg g^−1^). Even based on these conservative criteria, the study found 92 Ni hyperaccumulator taxa (65 known previously), 70 Mn hyperaccumulator taxa (11 known previously), 8 Co hyperaccumulator taxa (none known previously), and 5 Zn hyperaccumulator taxa (none known previously). This demonstrates that XRF screening of herbarium specimens has the potential to discover vast numbers of ‘new’ hyperaccumulator species in short surveys (lasting only a few months), even in well-studied floras such that of New Caledonia.

Our team is also currently developing a “Herbarium X-ray Fluorescence Spectroscopy & Digitalisation Station”. This mobile (cabinet-sized) system will permit ultra-fast herbarium digitisation (high-resolution optical image) in tandem with X-ray fluorescence spectroscopy analysis. We envisage that the instrument will have a high-flux X-ray source (50 Watt, Mo-source) and fast large area (150 mm^2^) Silicon Drift Detector (SDD) to permit very high throughput (~1000 specimens per 8-hr day). In addition to faster measurements, such a system would have the ability to measure a much wider range of elements, including lighter elements, such as macronutrients (*e.g*. Ca, K, P, S) that cannot be accurately quantified using current handheld XRF technology, thereby acquiring true ionomic profiles of analysed herbarium specimens. The concept is for the system to be deployed for dedicated projects at major global herbaria, prioritizing tropical herbaria in the Asia-Pacific Region, Africa and Central and South America.

To date, the potential to unlock foliar elemental information from plant herbarium collections has not been fully realised, but Herbarium XRF Ionomics may be transformative in the emerging field of ionomics and the discovery of hyperaccumulator plants. The demonstrations summarized here represent two of the largest individual contributions to the global inventory of hyperaccumulator plants, and include a substantial expansion in phylogenetic records, with many genera not previously known to contain hyperaccumulators. They also highlight the potential to uncover novel evolutionary, ecological, and biogeographic phenomena. This information could then be used to target specific species for genetic and ecophysiological experiments under controlled conditions. Such investigations can be approached with various other X-ray techniques, including synchrotron X-ray fluorescence microscopy (XFM), proton-induced X-ray emission (PIXE) and scanning electron microscopy with energy dispersive X-ray spectroscopy (SEM-EDS) to answer questions at every level of metal(loid) homeostasis in plants, from the rhizosphere interface, to uptake pathways in the roots and shoots^30^. Finally, this approach demonstrates a new value proposition for continued funding and retention of herbarium specimens globally^31^.

## Methods

### XRF instrument

The Niton XL3t 950 analyser (Thermo-Fisher Scientific; Fig. 1) uses a miniaturised X-ray tube (Ag anode; 6–50 kV, 0–200 µA max.) as its main excitation source. The X-ray tube irradiates the sample with a stable source of high-energy X-rays in a ~6 mm spot. Fluorescent X-rays generated from excitation by the incident beam are continuously detected, identified and quantified by the in-built Silicon Drift Detector (SDD) with an energy-resolution of ~185 eV at up to 60 000 counts per second. The instrument incorporates Compton Normalisation, appropriate for the relatively low elemental concentrations found in the low *Z* element bulk composition of plant material (compared to the high *Z* element matrix of ores or metallurgical samples). The XRF instrument produces X-rays in the form of the high-energy incident beam, and lower energy X-rays from scattering and fluorescence. It is important to limit exposure to these X-rays by correctly operating the instrument, and in most jurisdictions a Radiation User License is required to own and operate an XRF device.

### XRF calibration

A total of 590 dried plant samples were used from the first author’s field collections originating from Sabah, Malaysia^18^. The plant samples included known Co, Ni, Mn and Zn hyperaccumulator plants that were selected such that the dataset would cover element-specific concentration ranges ranging from ‘normal’ to ‘abnormal’ (*i.e*. hyperaccumulation). From each sample, a 6-mm diameter leaf disc (to match the approximate incident X-ray beam width) was extracted using a paper punch. A square plate of ~99.7% pure titanium (2 mm thick × 10 cm × 10 cm; Sigma-Aldrich 369489-90 G) was used behind the specimens to provide a uniform background. Before XRF testing, a sheet of cardboard with a square hole made to fit the titanium plate at the centre was fixed on the XRF table, and a sheet of herbarium mounting paper was placed over the titanium plate. All testing was conducted on this platform, ensuring that the beam window of the XRF analyser completely covered the leaf sample. XRF testing was carried out in the ‘Soils Mode’ for 60 seconds (a sensitivity analysis showed that after 60 s no further improvement in signal is obtained). After scanning, the leaf fragment samples were weighed and acid digested using 5 mL HNO_3_ (70%) and 1 mL H_2_O_2_ (30%) in a microwave digestion system (Milestone Start D) on a 45-minute programme^32^ and diluted to 30 mL with ultrapure water (Millipore 18.2 MΩ·cm at 25 °C) before analysis with ICP-OES (Varian Vista Pro II) for Ni, Co, Mn, Fe and Zn. Correction factors were derived by linear regression of XRF data against corresponding ICP-OES measurements.

### Herbarium XRF scanning

During six weeks, ~7300 herbarium specimens at the Forest Research Centre (FRC) in Sabah, Malaysia were measured with XRF, using the same settings and titanium backing plate described in the calibration procedure. Given that the FRC Herbarium contains ~350 000 specimens (~10 000 taxa) a selection was made and consisted of: all specimens in the families Phyllanthaceae, Salicaceae, Violaceae and Sapotaceae (totalling 5975 specimens), plus the genera *Walsura* (Sapindaceae – 98 specimens) and *Mischocarpus* (Meliaceae – 81 specimens), and a single specimen from all species (1183) known to occur on ultramafic soils in Sabah. Incompletely identified specimens were omitted. This selection was based on previous studies on hyperaccumulator plants in Sabah^18,33^ with the aim to target the most likely phylogenetic linages for hyperaccumulator plants.

In New Caledonia, herbarium XRF scanning was undertaken at the L’Institut de Recherche pour le Développement (IRD). The selection of specimens consisted of a number of families known to include hyperaccumulators, from which all available specimens (originating from ultramafic and non-ultramafic soils) were scanned. These were: Cunnoniaceae, Phyllanthaceae, Salicaceae, Sapotaceae and Violaceae. In addition, XRF scanning was undertaken on 1–4 (depending on availability) specimens selected from every dicot species known to occur on ultramafic soils in New Caledonia (on the basis of occurrence records and geological maps), totalling 1372 species^34^. Only dicotyledons were measured, and no gymnosperms or monocotyledons were considered in this study.
